# Total knee replacement plus physical and medical therapy or treatment with physical and medical therapy alone: a randomised controlled trial in patients with knee osteoarthritis (the MEDIC-study)

**DOI:** 10.1186/1471-2474-13-67

**Published:** 2012-05-09

**Authors:** Soren T Skou, Ewa M Roos, Mogens B Laursen, Michael S Rathleff, Lars Arendt-Nielsen, Ole H Simonsen, Sten Rasmussen

**Affiliations:** 1 Orthopaedic Surgery Research Unit, Aalborg Hospital, Aarhus University Hospital, Aalborg, Denmark; 2 Department of Health Science and Technology, Centre for Sensory-Motor Interaction, Aalborg University, Aalborg, Denmark; 3 Research Unit for Musculoskeletal Function and Physiotherapy, Institute of Sports Science and Clinical Biomechanics, University of Southern Denmark, Odense, Denmark; 4 Graduate School of Health Sciences, Aarhus University, Aarhus, Denmark; 5 Orthopaedic Surgery Research Unit, Aarhus University Hospital - Aalborg Hospital, Research and Innovation Center, 15 Soendre Skovvej, DK-9000, Aalborg, Denmark; 6 School of Medicine and Health, Aalborg University, Aalborg, Denmark

## Abstract

**Background:**

There is a lack of high quality evidence concerning the efficacy of total knee arthroplasty (TKA). According to international evidence-based guidelines, treatment of knee osteoarthritis (KOA) should include patient education, exercise and weight loss. Insoles and pharmacological treatment can be included as supplementary treatments. If the combination of these non-surgical treatment modalities is ineffective, TKA may be indicated. The purpose of this randomised controlled trial is to examine whether TKA provides further improvement in pain, function and quality of life in addition to optimised non-surgical treatment in patients with KOA defined as definite radiographic OA and up to moderate pain.

**Methods/Design:**

The study will be conducted in The North Denmark Region. 100 participants with radiographic KOA (K-L grade ≥2) and mean pain during the previous week of ≤ 60 mm (0–100, best to worst scale) who are considered eligible for TKA by an orthopaedic surgeon will be included. The treatment will consist of 12 weeks of optimised non-surgical treatment consisting of patient education, exercise, diet, insoles, analgesics and/or NSAIDs. Patients will be randomised to either receiving or not receiving a TKA in addition to the optimised non-surgical treatment. The primary outcome will be the change from baseline to 12 months on the Knee Injury and Osteoarthritis Outcome Score (KOOS)_4_ defined as the average score for the subscale scores for pain, symptoms, activities of daily living, and quality of life. Secondary outcomes include the five individual KOOS subscale scores, EQ-5D, pain on a 100 mm Visual Analogue Scale, self-efficacy, pain pressure thresholds, and isometric knee flexion and knee extension strength.

**Discussion:**

This is the first randomised controlled trial to investigate the efficacy of TKA as an adjunct treatment to optimised non-surgical treatment in patients with KOA. The results will significantly contribute to evidence-based recommendations for the treatment of patients with KOA.

**Trial registration:**

Clinicaltrials.gov reference: NCT01410409

## Background

Indications for total joint arthroplasty (TJA) vary greatly among countries [1]. Although patients considered eligible for TJA on average report more pain and functional limitation than patients not eligible for TJA, the overlap is substantial and no cut-offs can be established [2]. Adding a radiographic score to patient-reported pain and function does not improve identification of those considered eligible for TJA [2]. The incidence of total knee arthroplasty (TKA) in younger patients has rapidly increased [3,4] and only about every second patient considered eligible for TKA reported pain of about 50 or worse on a 0 to 100 scale [2], suggesting a broadening of indications to include also younger patients with less severe symptoms. A consensus on the indication for TKA in knee osteoarthritis (KOA) is in high demand, but requires the development of high quality evidence for the treatment options in KOA.

Evidence suggests that treatment of KOA should include multiple parallel treatment modalities [5,6]. On the basis of the existing evidence, clinical guidelines recommend a combination of patient education, exercise and weight loss as the first treatment option and that insoles and pharmacological treatment can be included as supplements [5-7]. Strong evidence suggests that exercise [8-11] and weight loss [12-14] reduce pain and improve functional level in patients with KOA. Furthermore, the evidence shows that patients with KOA undergoing patient education experience reduced pain and functional disability and improved wellbeing [10,15,16], while the evidence concerning insoles is conflicting, but still recommended [6,17,18]. Acetaminophen (paracetamol) reduces pain in KOA [19,20], and it is recommended as the analgesic of first choice [5-7]. When pain is insufficiently controlled with paracetamol, the addition of a second analgesic such as short term NSAID is recommended [5,6].

When non-surgical treatment is ineffective, TKA may be indicated [5-7]. The existing studies report improvement in pain and function following TKA [21]. However the quality of the evidence can be questioned since no RCTs have evaluated the efficacy of TKA compared with other treatment modalities [6]. Around 20 % of patients who receive a TKA experience little or no improvement in pain, disability and/or quality of life; a substantial proportion even develops chronic pain following TKA [22,23]. Hence, there is a need to further improve the treatment algorithm for KOA.

Optimisation of treatment may be done by combining the recommended non-surgical treatment modalities as a previous RCT suggests there may be an additive effect [13]. However, no one has yet investigated the combined effect of all the recommended non-surgical treatment modalities or the effect of an optimised non-surgical treatment combined with a TKA.

The purpose of this study is to examine whether TKA provides further improvement in quality of life, pain and function in addition to a 12-week evidence-based non-surgical treatment program in patients referred from primary care to an orthopaedic surgeon for evaluation of the need for TKA, with definite radiographic OA and no more than moderate pain.

We hypothesise that TKA, in addition to optimised non-surgical treatment, results in a significantly greater pain reduction, functional improvement and increase in quality of life at the 12-month follow-up in patients considered in need of TKA, with definite radiographic OA and a mean VAS pain score during the previous week of 60 or less.

## Methods/Design

### Study design

This is a randomised, assessor-blinded, controlled trial of TKA in addition to a 12-week multimodal, systematic non-surgical treatment (the MEDIC-treatment) with 12-month follow-up. Measurements will be taken at baseline, and 12, 26 and 52 weeks after the start of the MEDIC-treatment. The protocol conforms to CONSORT guidelines for parallel group randomised trials [24] and the protocol is designed to conform to the principles of the Declaration of Helsinki and has been approved by the local Ethics Committee of The North Denmark Region (N-20110024).

### Participants

The inclusion and exclusion criteria is selected to include patients considered to have a relative indication for TKA. This indication is defined as a knee condition considered by an orthopaedic surgeon to be in need of TKA, having definite radiographic KOA and having a mean Visual Analogue Scale (VAS) pain score during the previous week of 60 or less. This is in contrast to those considered to have an absolute indication for TKA defined as a report of a mean VAS pain score during the previous week of 60 or more.

We will recruit 100 patients meeting the following inclusion criteria:

1. Referred from primary care to an orthopaedic surgeon in a public hospital in The North Denmark Region for evaluation of the need for TKA;

2. Considered eligible for TKA by the surgeon;

3. Diagnosed with KOA using standing, weight-bearing knee radiographs (Kellgren-Lawrence score ≥2 on the original scale [25,26]); and

4. Aged ≥18 years.

The exclusion criteria are:

1. Bilateral simultaneous TKA;

2. Revision of prior TKA, unicompartmental knee arthroplasty or high tibial osteotomy;

3. Rheumatoid arthritis;

4. Mean pain the previous week > 60 mm on a 100 mm VAS;

5. Possible pregnancy or planning pregnancy;

6. Inability to comply with the protocol; and

7. Inadequacy in written and spoken Danish.

### Procedure

**Figure 1:**
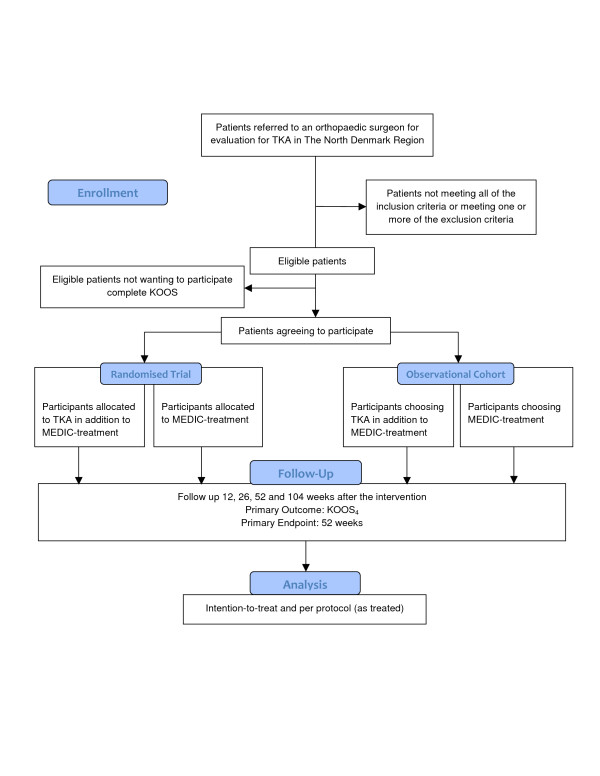
Flowchart.

The overall structure of the study is outlined in Figure 1. People in need of evaluation for TKA in The North Denmark Region are referred by their general practitioner to the outpatient clinics at Frederikshavn and Farsoe, Department of Orthopaedic Surgery, Aalborg Hospital, which specialise in performing TKA. A standardised weight-bearing antero-posterior knee x-ray is obtained [27].

In addition to written and verbal information, potential participants will watch a DVD as part of the recruitment process to standardise the information concerning the project. The DVD provides a description of the project in lay language and evidence-based information on what currently is known about KOA.

One of the orthopaedic surgeons at the outpatient clinics will assess potential participants against the inclusion criteria and exclusion criteria 1–3 and a research health worker assigned to the project will assess them against exclusion criteria 4–7. Informed written consent will be obtained from patients who are eligible and willing to participate. After the baseline measures are obtained, patients who agree to participate in the randomised controlled trial will receive their treatment assignment, either (i) TKA in addition to the MEDIC-treatment or (ii) the MEDIC-treatment only.

Participants will be reassessed after 12-weeks of MEDIC-treatment (12 week follow-up) and again after 6 months (26 weeks) and 12 months (52 weeks). In addition, there will be long-term follow-ups at two, five and ten years. Participants are asked to refrain from other treatments during the trial. All current medication use, co-morbidities and co-interventions will be recorded at all follow-ups.

### Randomisation procedure and concealment of allocation

The schedule for randomisation will be randomly generated using a computer before the initiation of the trial. The randomisation will be by random permuted blocks, stratified according to the clinic (Frederikshavn or Farsoe) to control for variation in patient characteristics in the two clinics. To conceal the outcomes of the randomisation, the allocation numbers will be put in concealed, opaque C5 envelopes. In blocks of eight, these envelopes will be placed in consecutively numbered opaque larger envelopes (seven larger envelopes in total for each clinic). An independent staff member will prepare the envelopes. These will be kept in a locked location accessible only by one research assistant at each of the respective clinics. Following the informed consent and completion of the baseline measures, a smaller envelope from the numbered larger envelopes will be opened by the research assistant and the allocation revealed to the participant. When only two smaller envelopes are left in the first of the numbered larger envelopes, the smaller envelopes of the second larger envelope will be added. When there are six smaller envelopes left in the sixth of the numbered larger envelopes at each clinic, the last two of the smaller envelopes will be added.

### Blinding

The outcome assessor will be blinded to group allocation, will not be involved in providing the interventions, and will be unaffiliated with the treatment sites. The participants and the project physiotherapist delivering part of the interventions cannot be blinded. The statistician performing the statistical analyses will be blinded to group allocation.

### The observational cohort study

Patients refusing to participate in the randomised controlled trial will be offered the option of participating in an observational cohort. The inclusion and exclusion criteria, study intervention, follow-up schedule, and study endpoints will be identical to the randomised controlled trial. The only difference is that the treatment will not be randomly assigned, because the participants will be able to choose between (i) TKA in addition to the MEDIC-treatment or (ii) the MEDIC-treatment.

Patients refusing to participate in both the randomised controlled trial and the observational cohort will be asked to fill out the Knee Injury and Osteoarthritis Outcome Score (KOOS) and report age and gender anonymously so as to improve the failure analysis.

### Interventions

#### Total knee arthroplasty

Surgery will be performed within 8 weeks from inclusion in the trial for all patients receiving a TKA in addition to the MEDIC-treatment. The surgery will be performed by the orthopaedic surgeon who assessed them during the recruitment phase at one of the two clinics.

A total cemented prosthesis with patellar resurfacing (NexGen CR-Flex fixed or LPS-Flex fixed, Zimmer, Warsaw, Indiana, USA) will be applied using standard methods [28].

Participants will be mobilised to sitting or standing within a few hours after surgery. Active and passive flexion and extension exercises following a standard protocol of The North Denmark Region will be initiated the day after surgery by a physiotherapist and continued once or twice daily during the hospitalisation phase, normally 2–3 days, to improve range of movement and muscle strength. After discharge, this treatment will be continued concurrently with the MEDIC-treatment supervised by the project physiotherapist.

#### The MEDIC-treatment

The MEDIC-treatment consists of five different interventions. Following the clinical guidelines, patient education, exercise and weight loss are the three core elements, while insoles and pharmacological treatment will be included when meeting objective test criteria and if considered needed by the treating clinician [5-7].

The participants allocated to, or choosing, TKA in addition to the MEDIC-treatment will start the MEDIC-treatment immediately after discharge from the hospital following their TKA (anticipated mean hospitalisation stay 3–4 days). The participants allocated to, or choosing, the MEDIC-treatment only will start the intervention right away. The MEDIC-treatment will take place at one site (Aalborg), located geographically between Frederikshavn and Farsoe.

#### Patient education

The purpose of the patient education in this study is to encourage the participant to actively engage in and take responsibility for the management and treatment of their KOA. The patient education is based on principles from The Osteoarthritis Management Course developed in Sweden (BOA, Gothenburg, Sweden), and consists of two sessions with a duration of 60 minutes each. The first session will focus on the diagnosis of KOA, the aetiology, symptoms and risk factors and a short overview will be given on the current treatment options of KOA. In the second session an in-depth description will be given of how KOA can be treated as well as guidance in self-help tools in relation to KOA. Both sessions will be held by the project physiotherapist in groups of up to 16 participants. The physiotherapist will facilitate discussion and interaction between the participants. Furthermore, the participants will receive a DVD containing all information that was provided during patient education.

#### Exercise

The NEuroMuscular EXercise training program for patients with osteoarthritis of the knee or hip who are scheduled for total joint replacement (NEMEX-TJR) will be applied in this study. It is a training program based on neuromuscular principles that has been found feasible in patients with severe hip or knee OA [29].

The duration of an exercise session is 60 min. and will be completed 2 times a week in the 12-week intervention period. The focus is on the affected leg, but exercises are carried out with both legs. The project physiotherapist heading the exercise is a specialist in the area and has participated in a course in NEMEX-TJR held by the originators. The participants will be under the supervision and instruction of the project physiotherapist and the training will also be supplied as images and text. The training will be class-based at Aalborg University Hospital (in classes of up to 8 participants). After 12 weeks of training it will shift to being home-based individual training, as the combination of class-based and individual home-based training has been shown to reduce pain more than home-based exercise alone [30]. Participants will be admitted continuously into the class so that the class consists of both novices as well as experienced participants. Each participant will be monitored individually to ensure that the training is tailored to the individual’s level of function and pain.

Pain is a significant problem for patients with KOA [31]. For that reason, the participants will be asked to monitor their pain during training in collaboration with the project physiotherapist using a VAS-scale. Pain up to 5 is “acceptable” during and after the exercise session. The morning after an exercise session, pain should subside to “pain as usual”. If pain does not subside, the intensity of the training will be reduced [32]. This pain monitoring system is part of the NEMEX program.

#### Diet

Participants with a Body Mass Index (BMI) ≥25 at baseline will be referred to a 12-week dietary weight loss program. The goal of the intervention is a reduction in body weight by at least 5 % and retention of this throughout the project period. The 5 % reduction in body weight is required to experience symptomatic relief [14]. The dietary intervention is carried out by the project dietician and is based on principles from Motivational Interviewing (MI) and consists of instruction and guidance in relation to diet. The participant’s readiness to change will be evaluated and forms the basis of the intervention. The focus is on getting the participant to take action regarding their diet using different strategies [33]. These strategies are supported by diet plans, written guides, recipes etc. depending on the needs of the individual participant. All participants referred to the project dietician will have four dietary sessions. The first session will take place in the first week of the intervention (60 minutes), while session 2–4 will take place 3, 6 and 11 weeks after the start of the intervention (30 minutes).

#### Insoles

The participants will receive one of two possible insoles depending on their hip-knee-foot alignment. The project physiotherapist will assess knee alignment using the single limb mini squat. The single limb mini squat has been found to be a valid and reliable tool when investigating medio-lateral motion of the knee in clinical settings [34]. Participants will be scored as having either a knee-medial-to-foot position (the knee moves medially to the 2^nd^ toe in three or more of five trials), a knee-above-foot position (the knee moves between the 2^nd^ and the 5^th^ toe) or a knee-lateral-to-foot position (the knee moves over or lateral to the 5^th^ toe).

1. Participants who score a knee-medial-to-foot or a knee-above-foot position will get an individually fitted non-wedged full length Formthotics System insole with medial arch support (Foot Science International, Christchurch, New Zealand).

2. Participants who score a knee-lateral-to-foot position will get an individually fitted 4 ° laterally wedged full length Formthotics System insole with medial arch support (Foot Science International, Christchurch, New Zealand).

The participants will be requested to use the insoles bilaterally in all shoes every day.

#### Medicine

In the case of no contraindications, paracetamol 1 g four times daily, ibuprofen 400 mg three times daily, and pantoprazol 20 mg daily will be prescribed for use during the intervention period. The prescription will be renewed every three weeks in order to supervise the use of, and indications for, medication. If the patient experiences pain relief, making them question continuation of the prescription during a three-week period, they will be urged to contact the research physiotherapist who will record the discontinuation of medication.

#### Booster sessions after the 12 weeks of MEDIC-treatment

Following the 12-week MEDIC-treatment, the participants will be encouraged to continue the MEDIC-treatment unsupervised at home with the same frequency as during the 12-week intervention. There will be a transition period of 8 weeks where the participants will exercise in class and at home alternately, and two additional 30-minute telephone sessions with the project dietician (26 and 39 weeks after the start of the MEDIC-treatment) will be scheduled. Furthermore, the participants will be contacted by telephone by the project physiotherapist 8 times in the interval between the transition period and the 12-month follow-up to ensure a higher compliance to the MEDIC-treatment. These initiatives have previously been found to be effective, even in relation to long-term outcomes [13,35,36].

### Crossovers

Crossovers are a common problem in studies randomising to operative or non-operative treatment [37,38]. To minimize the number of crossovers, the following initiatives are used: Participants randomised to both MEDIC-treatment and TKA will exercise together and those with MEDIC-treatment only will be in their own class. At the same time, the project physiotherapist and project dietician will be trained in retention of the participants in their respective groups based on experience from previous studies [13,35,39-45].

Participants who experience impairment of their symptoms will be reassessed by the orthopaedic surgeon who assessed them in the recruitment phase. Pre-defined criteria for crossover to TKA or revision of TKA are a score for quality of life and/or for pain equal to or below 25 on the KOOS and agreement between the participant and the orthopaedic surgeon that a TKA or revision of a TKA is necessary.

The reason for each crossover will be registered. Participants crossing over will remain in the study and analysed in a intention-to-treat analyses.

### Baseline data

Gender, age, nationality, height, alcohol intake, smoking habits, duration of KOA symptoms, previous injuries, treatment and use of medication regarding the affected knee, co-morbidities, physical activity and exercise, preferred treatment, previous arthroplasty, living arrangement, satisfaction with self-management of pain, education level and employment status, income, home help, and the short version of the Hip/Knee Osteoarthritis Decision Quality Instrument (HK-DQI)[46] will be obtained by questionnaire. After the randomisation, the participant will be asked about their belief in the effect of the assigned/chosen treatment in relation to pain, function and quality of life. The radiographic severity of KOA will be assessed on the baseline x-ray using the Kellgren and Lawrence grading system [25].

### Primary outcome measure

The primary outcome will be the change from baseline to 12 months in the average score for four of the five Knee Injury and Osteoarthritis Outcome Score subscales covering pain, symptoms, activities of daily living, and quality of life (KOOS4), with scores ranging from 0 (worst) to 100 (best) (Table 1) [47,48].

**Table 1:** Study measures

**Construct assessed**	**Data collection instrument**	**Time of collection**
**Primary outcome measure**		
Pain, symptoms, physical function and QOL	Average score of four of the KOOS subscales, KOOS_4_	0, 12, 26 and 52 weeks
**Secondary outcome measures**	**Data collection instrument**	**Time of collection**
	*PROMs*	
Pain, symptoms, ADL, QOL and Sport & Rec	The five individual subscales of KOOS	0, 12, 26 and 52 weeks
Health outcome	EQ-5D-3 L	0, 12, 26 and 52 weeks
Self-efficacy in improving pain, function and QOL	100 mm VAS	0, 12, 26 and 52 weeks
Pain intensity in various situations	100 mm VAS	0, 12, 26 and 52 weeks
Pain location	Region-divided body chart	0, 12, 26 and 52 weeks
	*Objective measures*	
Functional performance	Timed Up and Go	0, 12, 26 and 52 weeks
Functional performance	20-meter walk test	0, 12, 26 and 52 weeks
Weight change	Percentage-wise change in weight from baseline to follow-up	0, 12, 26 and 52 weeks
Muscle strength	HHD - maximum isometric strength in flexion and extension	0, 12, 26 and 52 weeks
Pain reactions	Handheld algometer – PPTs at four sites in the peripatellar region and at m. tibialis anterior	0, 12, 26 and 52 weeks
**Other measures**	**Data collection instrument**	**Time of collection**
Compliance with exercise	Treatment records, log-book	Continuously
Use of medication	Questionnaire	0, 12, 26 and 52 weeks
Compliance with diet, insoles and patient education	A five-point scale (ranging from never to all the time)	0, 12, 26 and 52 weeks
Satisfaction	A five-point Likert scale	0, 12, 26 and 52 weeks
Adverse events	Treatment records, hospital records and questionnaire	Continuously
Health and non-health care costs	Hospital records and questionnaire	0, 12, 26 and 52 weeks

PROMs = Patient-reported outcome measures, QOL = quality of life, ADL = activities of daily living, Sport & Rec = sports and recreational activities, HHD = Handheld Dynamometer, PPTs = Pressure pain thresholds.

### Secondary outcome measures

Several other patient-reported outcome measures will be used (Table 1). The five subscales of KOOS (the fifth scale being difficulty in sports and recreational activities) [47,48], the EQ-5D-3L for economic appraisal [49], and self-efficacy in relation to reducing pain and increasing function and quality of life using a 100 mm VAS with terminal descriptors of ‘very unsure’ and ‘very sure’ will be used. Furthermore, pain intensity will be measured on a 100 mm VAS with terminal descriptors of ‘no pain’ and ‘worst pain possible’ in the following situations: at rest, after 30 min. of walking and worst pain and least pain in the previous 24 hours. The participants will be asked to shade regions where they have had pain during the previous 24 hours on a region-divided body chart.

A number of objective measures will be assessed (Table 1). Prior to the start of the study, the outcome assessor will undergo a period of supervised training in how to use the objective measures to optimise the reliability of the measurements. To retain the blinding of the assessor, all participants will be wearing a loose sticking plaster on both knees at all follow-ups to cover the area were a possible scar from the surgery would be. A Timed Up and Go [50] and 20-meter walk test [51] will be used as measures of the functional performance of the participants. Percentage change in weight from baseline to follow-up is another objective measure which will be used in this study. The participant’s weight will be measured without shoes at the same time of day and on the same scale (seca 813, seca gmbh & co. kg., Hamburg, Germany) at baseline and at all follow-ups.

Maximum isometric muscle strength will be measured in knee flexion and knee extension bilaterally in a make test using a handheld dynamometer (HHD), the Powertrack II^TM^ Commander (JTech Medical Industries, Salt Lake City, Utah, USA). The participant will be seated on an examination couch with their hip in a 90 ° flexion position and instructed in stabilising themselves by holding onto both sides of the couch. The HHD will be fastened to the couch (knee extension) or a wall bar in front of the participant (knee flexion) with a strap keeping the HHD just proximal to the lateral malleolus, perpendicular to the limb being tested, to ensure that the knee is kept in a 75° angle during the test, a starting position used in previous studies when measuring isometric strength in KOA [52,53]. The participant will be asked to carry out a 5-second isometric maximum voluntary contraction (MVC) pushing against the dynamometer and the hand of the examiner. The assessor will use the standardised cue “*push … hold, hold, hold and relax*” and encourage the participant to do their best. To ensure that the participant has understood the test procedure, he or she will be asked to perform a sub-maximal test and then an MVC before starting the measurement. The highest value of four consecutive measurements and the mean of the three highest values will be used in the analysis for both knee extension and knee flexion. The participant will be given a 30-second rest between each measurement.

**Figure 2:**
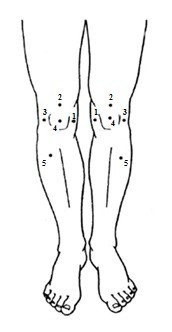
PPT measurement sites.

To assess pressure pain thresholds (PPTs), a hand-held pressure algometer (Algometer Type II, Somedic AB, Hoerby, Sweden) with a 1 cm^2^ probe will be used. The probe will be placed perpendicular to the skin and force applied at a constant rate of 30 kPa/s until the participant defines the pressure as pain and presses a button. PPTs will be assessed bilaterally at one control site on m. tibialis anterior (5) (5 cm distal to the tibial tuberosity) and four sites in relation to bony landmarks in the peripatellar region, 3 cm medial to the midpoint of the medial edge of the patella (1), 2 cm proximal to the superior edge of the patella (2), 3 cm lateral to the midpoint of the lateral edge of the patella (3) and at the centre of the patella (4) (Figure 2). Before starting the measurement, the test is performed once or more on m. extensor carpi radialis longus to make sure that the participant has understood the test procedure. A PPT will be obtained twice from each site and the mean of the two measurements will be used in the statistical analysis [54]. The participant will be asked about the location and type of their knee pain using the interviewer-administered questionnaire Knee Pain Map, which has been found reliable for this purpose [55].

The test setup for both isometric muscle strength and PPTs will be investigated in a test-retest reliability study on 20 participants.

### Other measures

A number of other measures will be obtained in this study (Table 1). Compliance with exercise will be monitored by the physiotherapist during the 12 weeks. Compliance is measured as the total number of exercise sessions completed out of the expected 24 sessions (two sessions a week over twelve weeks). Good compliance is defined as participation in 75 % or more of the exercise sessions, medium compliance as participation in 50-74 % of the sessions and poor compliance as participation in less than 50 % of the sessions. Following the 12-week intervention, the participants will be requested to record their weekly training to investigate the long-term compliance. The participants’ use of medication will be recorded in a medication diary, which will be examined as part of the follow-up. Compliance with the other aspects of the MEDIC-treatment will be assessed at each follow-up, using a five-point scale assessing the adherence to the treatment (never, every month, every week, every day, all the time).

At each follow-up, the participants will be asked to rate their satisfaction with the treatment so far on a five-point Likert scale.

Adverse and seriously adverse events will be registered in three ways and divided into index knee or sites other than index knee. The project physiotherapist will record any adverse events that the participant experiences or tells them about. For the participants allocated to, or choosing, TKA, a project worker will look through hospital records to register if any pre-defined perioperative and postoperative adverse events occurred. At all follow-ups, the assessor will use open-probe questioning to assess adverse events in all participants (Table 2).

**Table 2:** Adverse events

**Method of registration**		**Adverse event**
Treatment records		
		All events reported by the participant
Hospital records		
	*During surgery*	
		Patella fracture
		Tibia fracture
		Femur fracture
		Rupture of the patella tendon
		Other
	*Postoperatively*	
		Deep infection
		Surgery demanding skin necrosis
		Surgery demanding scar tissue adherences
		Thrombophlebitis in demand of anticoagulant treatment
		Patella sub-/luxation
		Supra-condylar femur fracture
		Permanent n. peroneus paresis
		Pulmonary embolism
		Patella fracture
		Aseptic loosening
		Polyethylene defect (tibia)
		Polyethylene defect (patella)
		Secondary insertion of patella component
		Instability
		Pain without loosening
		Other events related to the index knee
		Other events not related to the index knee
Questionnaire		
		All events reported by the participant using open-probe questioning

Information on direct health care costs and direct non-health care costs will be collected retrospectively and at all follow-ups. Direct health care costs will include costs of all elements in the perioperative and postoperative period (hospitalisation, surgery, medication, additional health provider visits, etc.) in participants undergoing a TKA (in both groups), cost of the MEDIC-treatment and compliance with the treatment. These aspects will be valued using published prices for medical costs in Denmark. Direct non-health care costs will include number of days lost from work, working shorter hours, sick pay or income (if relevant) and change in home help.

### Sample size

Based on the primary outcome KOOS_4_ at the primary endpoint after 12 months, we expect that the group allocated to TKA in addition to the MEDIC-treatment will improve at least 10 points more than the group allocated to MEDIC-treatment alone. Using a common between-subject standard deviation of 14 sample size calculations show that 41 participants in each group are required to detect a statistical difference (power of 90 % and significance level at 0.05 (two-sided)). To account for crossovers and missing data, the drop-out rate will be set to 20 % and therefore, a total of 100 participants will be randomised.

### Statistical analysis

The primary outcome measure is KOOS_4_ 12-month follow-up. Following an intention-to-treat approach, statistical analysis will be based on a generalized estimating equations regression model for KOOS_4_ score at all follow-up times in order to take into account the repeated measurements on the patients. The model will incorporate the effects of treatment, follow-up time, treatment-by-follow-up time interaction, and KOOS_4_-score at baseline. Secondary analyses will assess heterogeneity between sites. Treatment compliance will be correlated to the outcome in order to investigate if compliance is associated with the outcome.

### Economic evaluation

The economic evaluation will be based on the Danish healthcare system. It will be conducted as a cost-utility analysis estimating the ratio between the direct health and non-health care costs (corrected for cost of funding) and quality-adjusted life years (QALYs). QALYs will be calculated using changes in EQ-5D from baseline to the 12-month follow-up.

### Timelines

Ethics approval was obtained from The North Denmark Region in June 2011. Recruitment and training of the involved project physiotherapist and dietician was undertaken in July and August 2011 and recruitment of participants started September 2011. All participants are expected to have completed the 12-month follow-up by December 2013.

## Discussion

There is a lack of high quality evidence concerning the efficacy of TKA [6] and around 20 % of KOA patients experience little or no improvement in pain, disability and quality of life following the TKA [22]. Therefore an evaluation of the efficacy of TKA compared with other treatments of KOA is essential.

There are several strengths of the design of this study. Firstly, this will be the first study assessing TKA in the treatment of KOA in a randomised, controlled design that can evaluate the additional effect of a TKA to the recommended non-surgical treatment.

Secondly, the recruitment of participants and multimodal approach to the non-surgical treatment resembles contemporary examination and treatment of KOA in Denmark and several other countries. Given that the treatments applied in this study alone are recommended and, for most of them, well documented in the treatment of KOA [5,6], we contend that the combination of treatments would be even more efficacious.

Thirdly, the treatment of the participants in this study following the preliminary assessment will be individualised within the possibilities of a randomised controlled trial framework. In this way, it will be possible to target the treatment on the basis of the characteristics of the individual, according to the recommendations of tailored treatment in treatment guidelines [5,7]. The semi-structured nature of the MEDIC-treatment will enable the project physiotherapist and dietician to tailor the treatment to the individual within the predefined protocol, which, on the other hand, will reduce treatment variation and enhance the reporting of the study and its replicability.

The multimodal approach in the MEDIC-treatment could be seen as a limitation since it will be impossible to identify the efficacy of the different treatments alone. However the purpose of this study is not to determine the effect of non-surgical treatments compared with each other, but to evaluate the additive effect of TKA. For this purpose, the design of the study can be considered optimal, since the MEDIC-treatment is a combination of recommended treatment modalities given to both groups [5,6].

The outcome measures of this study include patient-reported outcome measures of pain, function, quality of life, health outcome and self-efficacy. A range of objective outcome measures are included to incorporate functional performance, strength, change in weight, and pressure pain thresholds. The goal of the treatment is to improve function and quality of life and reduce pain. The objective outcome measures allow the investigation of the underlying mechanisms that may explain changes in pain and function. Furthermore, a health economics assessment is included to evaluate the treatment in relation to cost-effectiveness and implementation.

## Conclusions

We have designed this study as a randomised controlled trial to investigate if TKA plus a 12-week multimodal, optimised non-surgical treatment is more efficacious and cost-effective than a 12-week multimodal, systematic non-surgical treatment only in patients with KOA. Since it is the first study evaluating TKA in a randomised controlled trial, the results will enable evidence-based recommendations for the treatment of patients with KOA.

## Competing interests

The authors declare that they have no competing interests.

## Authors’ contributions

STS is leading the co-ordination of the trial. STS, EMR, MBL, MSR, LAN, OS and SR assisted with the protocol design and procured the project funding. STS wrote this manuscript. All authors participated in the trial design, provided feedback on drafts of this paper and read and approved the final manuscript.

## Pre-publication history

The pre-publication history for this paper can be accessed here:

http://www.biomedcentral.com/1471-2474/13/67/prepub
